# The content of diagnostic information has an immediate effect on pain with loading in people with midportion Achilles tendinopathy: a randomized clinical experiment

**DOI:** 10.1016/j.bjpt.2025.101244

**Published:** 2025-07-12

**Authors:** Nigel J Travers, Mervyn J Travers, William Gibson, James R Debenham, Dana A Hince, Benedict M Wand

**Affiliations:** a Star Physiotherapy, West Perth, WA, Australia; b School of Health Sciences, The University of Notre Dame Australia, Fremantle, WA, Australia; c Majarlin Kimberley Centre for Remote Health, The University of Notre Dame Australia, Broome, WA, Australia; d Institute for Health Research, The University of Notre Dame Australia, Fremantle, WA, Australia

**Keywords:** Diagnosis, Musculoskeletal pain, Pain perception, Predictive processing, randomized controlled trial, Tendinopathy

## Abstract

**Background:**

Contemporary models of pain suggest the beliefs an individual holds regarding the cause of pain may influence pain intensity. Direct evidence supporting this idea is lacking in clinical populations.

**Objective:**

Investigate if the content of diagnostic information about the cause of pain influences pain and function in people with Achilles tendinopathy.

**Methods:**

Fifty recreational runners with Achilles tendinopathy participated in this randomised clinical experiment. Participants in the experimental group received an explanation of tendon pain that highlighted changes in muscle function as the primary problem, without reference to tendon pathology. Participants in the control group received an explanation of tendon pain that prioritised tendon pathology. The primary outcome measure was maximal pain intensity during a standardised hopping task measured on a 0–100 visual analogue scale. Secondary outcomes were lower limb stiffness while hopping and time in seconds for pain to ease upon completion of the hopping task.

**Results:**

Pain intensity was lower in the experimental group post intervention (mean difference = 12.3 mm; 95 % CI: 3.2, 21.5). Lower limb stiffness was higher in the experimental group (mean difference = −1546 Nm^−1^; 95 % CI: −3296, 204) and time-to-ease was near identical (mean difference = 0 s; 95 % CI: −11, 11).

**Conclusion:**

The content of diagnostic information influenced pain intensity with loading in people with Achilles tendinopathy. The effect on lower limb stiffness was uncertain and there appears to be no effect on time-to-ease.

## Highlights

• The content of diagnostic information influenced pain intensity with loading in people with Achilles tendinopathy.
• The effect on motor function was uncertain, though the changes observed might indicate decreased confidence in loading the tendon in those given a pathology orientated explanation.
• The results of this study should prompt clinicians to carefully consider the language they use when discussing the factors contributing to the clinical condition in people with Achilles tendinopathy.

## Introduction

Contemporary views of pain highlight the importance that beliefs about the body in pain have on pain intensity and associated disability,^1^, ^2^, ^3^, ^4^, ^5^ particularly beliefs that might drive the individual to predict pain with action.^6^ One key source of these beliefs comes from the diagnostic information about the cause of pain provided by health care practitioners.^7^ It is common for diagnostic models of musculoskeletal pain conditions to highlight various aspects of tissue damage or pathology as particularly important contributors to the pain experience.^8^, ^9^, ^10^ These types of diagnostic models have been shown to influence people’s perception of the seriousness of a particular clinical condition^11^^,^^12^ and may foster a formulation of the body as fragile and in need of protection, plausibly contributing to heightened sensitivity and excessively protective behaviours.^5^^–^^7^

There is a wealth of information showing that manipulation of people’s views and beliefs changes pain.^13^ In particular, there is evidence that manipulating how the body is viewed influences the pain experience in experimentally-induced pain.^14^^,^^15^ There is also indirect evidence suggesting that diagnostic information about the state of the body might influence outcome in clinical populations.^16^^–^^18^ We were interested in directly exploring this idea in a clinical pain population, particularly the immediate impact diagnostic information regarding the state of the body might have on sensitivity and motor function. Our focus was on immediate effects as later changes in pain and function would be confounded by actions and interventions enacted after the exchange of diagnostic information.

Achilles tendinopathy (AT) is a common musculoskeletal pain condition well-suited to exploring the immediate effects of diagnostic information on pain and function. Tissue pathology is frequently referenced in explanatory models of this condition.^8^^,^^19^ Pain is consistently and easily reproduced with repetitive application of lower limb stretch shortening cycle activities,^20^ which are a core feature of lower limb function. A composite measure of lower limb stiffness provides a simple estimate of stretch shortening cycle capacity, and stretch shortening cycle capacity has been shown to be impaired in people with AT.^21^

To help understand the impact diagnostic information has on clinical status, this study aimed to investigate if a diagnostic explanation that did not reference tissue pathology had a different effect on pain and motor function than a traditional diagnostic explanation that included reference to tissue pathology as an important contributor to the pain experience. Our hypothesis was that both pain intensity and protective motor responses would be greater in people provided with a traditional pathology orientated diagnostic explanation.

## Methods

### Design

This study received institutional ethical approval from The University of Notre Dame Australia Human Research Ethics Committee (reference number: 019061F) and all participants gave written informed consent before data collection began. We undertook a prospectively registered, parallel, two-arm, randomised clinical experiment with concealed allocation, participant and assessor masking, and intention-to-treat analyses. A random number sequence was computer-generated by a researcher not involved with recruitment, and concealment was ensured by sealing allocations in opaque, consecutively numbered envelopes. To ensure masking and control for demand characteristics^22^ participants were not made aware that they were participating in a controlled experiment until after data collection were complete. The researcher calculating the pain intensity scores and lower limb stiffness values was masked to group allocation and all analyses were undertaken by a masked statistician. A single musculoskeletal physical therapist provided both experimental and control explanations and was not masked.

### Participants

Participants were recruited through a group of private physical therapy practices in Perth Western Australia. The inclusion criteria were recreational runners with a history of unilateral mid-portion AT for over one-month and a Victorian Institute of Sports Assessment–Achilles (VISA-A) score below 80. Participants were excluded if they had: insertional AT; ceased running altogether as a result of this episode of Achilles pain; received previous care for this episode of Achilles pain; a non-painful hop test; a history of foot or ankle injury within the previous six-months; any coexisting lower quadrant musculoskeletal problem; any report of low back pain requiring consultation within the previous three-months.

### Initial assessment

People presenting to the clinic of the first author with an initial presentation of Achilles tendon pain were considered as potential study participants. Prior to randomisation all potential participants provided demographic and training related information and underwent a full clinical examination by a senior physical therapist to establish the diagnosis of mid-portion AT and confirm other eligibility criteria. At this point, eligible participants had all testing procedures explained and were invited into the study. Willing participants then provided signed consent. All participants were told the study was simply an investigation into the stability of lower limb stiffness with hopping over time and they would be asked to undertake two hopping tests 20 min apart. They were debriefed as to the true nature of the study after the second testing session.

To familiarise them to the task, participants were shown a demonstration of the hopping protocol and were then asked to perform 10 self-paced sub-maximal hops with their unaffected leg on a mobile timing mat. After a two-minute break formal baseline testing on the affected leg commenced. Participants were told they would be asked to rate the maximal pain intensity they experienced while hopping immediately afterwards, and that the time for their pain to ease back to baseline levels would also be assessed by them using a stopwatch. Next, participants rated their *expected* maximal pain intensity with the hopping protocol and then performed 10 self-paced hops on their affected leg. On completion of the 10 hops, maximal pain intensity and Time-to-Ease were recorded (see below for details).

### Assessment post intervention

At this point, the therapist left the room and opened the next sequentially numbered envelope, and the participant was assigned to either the experimental diagnostic explanation or the control diagnostic explanation. The therapist then re-entered the room and provided participants with the allocated diagnostic explanation for their condition, as per usual clinical practice (see below for details). Upon completion of the diagnostic explanation, post intervention data were collected using the same hopping protocol as outlined above. Both interventions were matched for time to ensure that the time between the pre and post hopping assessments was the same for both groups.

### Interventions

#### Experimental condition: tendon sensitivity as a result of functional problems within lower limb musculature

Participants in the experimental group were informed that their problem was Achilles tendinopathy and were told their Achilles tendon sensitivity was related to functional problems within the musculature of the lower limb and subsequent changes in the way the tendon was loaded during the stretch shortening cycle. The diagnostic explanation acknowledged that the tendon was abnormally sensitive but made no reference to tendon pathology or any aspect of tendon structure that might suggest damage or fragility. The essence of the messaging was that the tendon was sensitive as a consequence of problems in lower limb function – the concept of the tendon as a victim of problems elsewhere, not the primary culprit. A standardised script was developed to ensure consistency in messaging, with some scope for individualisation of information (see Supplementary material). A series of images of athletes performing normal stretch shortening cycle activities was included to match the use of images within the control condition. Participants were invited to ask questions in relation to this explanation throughout the period of information sharing.

#### Control condition: tendon sensitivity as a result of structural changes within the tendon

Participants in the control group were informed that their problem was Achilles tendinopathy and were provided with a more traditional explanation for their Achilles tendon sensitivity, based on the continuum of tendinopathy.^8^ The essence of the messaging for this group was that the tendon was sensitive as a consequence of structural alterations within the tendon. Reference was made to contribution from functional problems within the lower limb musculature, however tendon pathology was presented as central to the abnormal sensitivity state. A standardised script which allowed some scope for individualisation was also employed (see Supplementary material) and a series of images of an Achilles tendon were used to visualise the stages of tendinopathy, from an acute reactive phase to a chronic degenerative tendon.^8^ The images that corresponds to the participant’s current timeline was pointed out and discussed in greater detail. Questions were similarly invited throughout the explanation.

### Outcome measures

#### Primary outcome

##### Pain intensity

The primary outcome was maximal pain intensity experienced during the performance of 10 self-paced hops. A 0–100 visual analogue scale (VAS) anchored 0 = no pain and 100 = extreme pain^23^ was presented to the participant immediately on completion of the hopping protocol. Participants were asked to rate their Achilles pain by placing a vertical line at the point on the scale that best corresponded to their maximal pain intensity. Participant responses were converted to a number by measuring the distance in millimetres from their mark to the left anchor by an independent assessor masked to condition.

#### Secondary outcomes

##### Lower limb stiffness

Vertical leg stiffness during hopping was estimated using a spring mass model.^24^ A portable timing mat (PASPORT 2-Axis Force Platform, PS – 2142, PASCO scientific, Roseville, California) recording at 200 Hz was used to measure flight time and contact time for every hop. All hopping data were captured using PASCO Data Studio software (1999–2011©). Raw data were exported to a custom-built program (LabVIEW, National Instruments, Version 8.2.1, U.S.) and filtered using a second-generation low-pass Butterworth filter with cut-off frequency of 50 Hz. The first and last ground contacts were removed to account for the fact that the first hop begins from a standing start and the last contact phase represents a landing strategy. Therefore, the mean stiffness value of hops two to nine was calculated. A member of the research team masked to group allocation undertook all data filtering and calculated the leg stiffness values using a previously validated method.^24^

##### Time-To-Ease

We calculated the time in seconds it took for pain to return to baseline levels post-hopping. The therapist started a timer immediately on conclusion of the 10 hops, the participant was positioned in sitting and given the timer and instructed to press *stop* when their pain had returned to the baseline level. If the participant’s pain had not eased to baseline within 15 min, Time-to-Ease was recorded as 900 s.

#### Process variable

##### Expected pain intensity

To provide insight into the influence of expectation on the primary outcome, a 0–100 visual analogue scale (VAS) anchored 0 = no pain and 100 = extreme pain was presented to the participant immediately prior to each hopping test. Participants were asked to rate their *expected* maximal Achilles tendon pain intensity with the hopping protocol by placing a vertical line at the point on the scale that best corresponded to their *expected* maximal pain intensity. Participant responses were converted to a number by measuring the distance in millimetres from their mark to the left anchor by an independent assessor masked to condition.

### Sample size calculation

There is no consensus and little data on which to base an estimate of smallest worthwhile effect for clinical experiments of this kind. We powered the study to detect a *moderate effect* of a 15 mm between group difference in maximal pain intensity based on guidance on interpreting between-group differences in clinical trials from the American College of Physicians Clinical Practice Guideline for Low Back Pain (10–20 points on a 100-point VAS).^25^ A standard deviation of 19 was assumed based on data obtained by our group from an unpublished pilot experiment (*n* = 14) using the same hopping protocol. Power was set at 80 % and significance at 5 % and we used the method of Borm et al,^26^ to calculate the sample size required. This resulted in a sample size estimate of 38. We oversampled by 12 in case of unusable data and the uncertainty of assumptions, giving a final sample size of 50.

### Data analysis

The analyses were conducted using STATA version 17 (StataCorp LLC, TX, USA). All participants were analysed as per their assigned group. Descriptive statistics were used to describe demographic and clinical characteristics of the sample. The effect of group assignment was tested using separate regression models appropriate to the distribution, after adjustment for the corresponding values pre-intervention. Specifically, expected and experienced pain intensity used a Tobit model, lower limb stiffness a linear regression model, and Time-to-Ease used median (quantile) regression. Group differences are reported with 95 % confidence intervals.

## Results

### Compliance with trial protocol

Our recruitment target was met, and all enrolled participants met eligibility criteria. All participants received the intervention to which they were allocated. All outcome measures outlined in the registered protocol are reported. The analysis was conducted as per the trial pre-registration.

### Flow of participants through the study

**Fig. 1:**
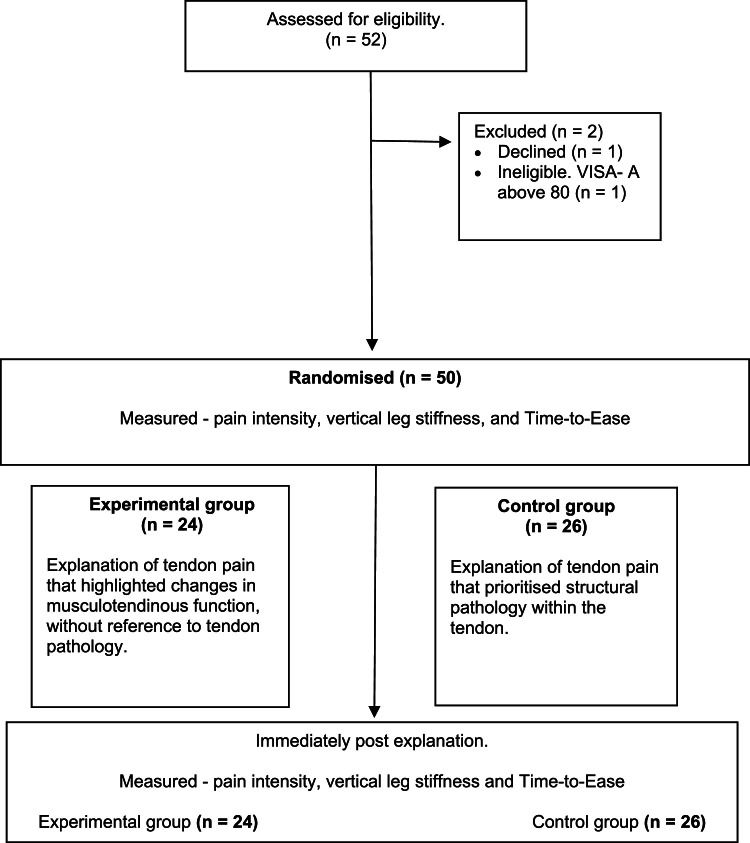
Flow of participants through the trial.

Recruitment occurred between June 2020 and October 2021. Fifty-two potential participants were assessed for eligibility into the study, 50 participants met all criteria and agreed to participate. FIG. 1 shows the flow of participants through the study. All participants completed the experiment and primary outcome data were obtained for all 50 participants. Stiffness data were not calculable from two participants in the control group and one participant in the experimental group due to technical problems with the timing mat. No missing data were imputed. All participants provided Time-to-Ease data. There were no adverse effects reported in the post-study debrief. The demographic and clinical characteristics of participants in each group can be found in Table 1**.**

**Table 1:** Baseline characteristics of the study participants.

Characteristic	Randomised (*n* = 50)	
	Experimental group (*n* = 24)	Control group (*n* = 26)
Age *(years)*, mean (SD)	45 (11)	43 (9)
Sex n male (%) n female (%)	17 (71) 7 (29)	14 (54) 12 (46)
Painful side, n right leg (%)	14 (58)	14 (54)
Dominant leg, n right leg (%)	21 (88)	23 (88)
Mean run frequency per week (SD)	3 (1)	3 (1)
Median run distance in km per week (IQR)	18 (12 to 35)	14 (10 to 30)
Height (cm), mean (SD)	177 (8)	176 (7)
Body mass (kg), mean (SD)	77 (13)	77 (14)
Pain duration (weeks), median (IQR)	38 (9 to 104)	29 (10 to 150)
VISA-A total score, mean (SD)	56 (13)	55 (13)

IQR, interquartile range; SD, standard deviation; VISA-A, Victorian Institute of Sport Assessment -Achilles.

### Process variable: predicted pain intensity

Predicted pain intensity post-intervention was slightly less in the experimental group with an adjusted mean difference of 0.6 mm (95 % CI: −8.8, 9.9).

### Primary outcome: actual pain intensity

Actual pain intensity post-intervention was less in the experimental group with an adjusted mean difference of 12.3 mm (95 % CI: 3.2, 21.5).

### Secondary outcomes: stiffness and time-to-ease

Vertical leg stiffness post-intervention was higher in the experimental group with an adjusted mean difference of −1546 Nm^−1^ (95 % CI: −3296, 204). The Time-to-Ease post-intervention was near identical between groups with an adjusted median difference of 0 s (95 % CI: −11, 11). A summary of all data can be found in Table 2 and a graphical representation can be found in Supplementary material.

**Table 2:** Pre and post intervention values by group, difference within groups, and point estimates with 95 % confidence interval for difference between groups.

Outcome	Groups				Difference within groups		Difference between groups
	Pre-Intervention		Post-Intervention		Pre minus Post Intervention		
	Exp (*n* = 24)	Con (*n* = 26)	Exp (*n* = 24)	Con (*n* = 26)	Exp	Con	Exp minus Con post intervention^a^
Predicted pain intensity (0–100)	42.2 (22.4)	42.3 (21.1)	36.3 (24.2)	37.0 (20.9)	−6.0 (17.1)	−5.4 (17.4)	0.6 (−8.8, 9.9)
Experienced Pain intensity (0–100)	33 (24.6)	32.4 (24.0)	25.4 (24.3)	36.7 (28.1)	−7.6 (17.4)	4.4 (15.6)	12.3 (3.2, 21.5)
Vertical leg stiffness (Nm^−1^)^b^	11,917 (4526)	11,095 (3142)	12,178 (3823)	10,280 (3213)	261 (4886)	−814 (2394)	−1546 (−3296, 204)
Time-to-Ease (Seconds)	11 (10–43)	13 (2–47)	10 (2–43)	10 (2–40)	−2 (−10 to 0)	0 (−16 to 4)	0 (−11, 11)

Con, control group; Exp, experimental group; Nm^−1^, Newton metres per second.

Pain intensity and stiffness data are reported as mean (SD). Time-to-Ease data are reported as median (IQR).

a Between group differences (95 % confidence interval) estimated from the regression model after adjusting for pre intervention values.

b Data missing from 3 participants due to equipment failure.

## Discussion

The aim of this study was to investigate if the content of diagnostic information about the cause of pain influenced pain and motor function in a clinical pain population. People who presented for their initial consultation with an Achilles tendon problem were randomised to receive either a diagnostic explanation that did not reference tissue pathology or a traditional diagnostic explanation that included reference to tissue pathology as a contributor to the pain experience. Study participants and assessors were masked, and we controlled for demand characteristics by conducting the study within the usual clinical context and ensuring that participants were unaware that they were involved in a controlled experiment.^22^ Indeed, it might be argued that suggestion effects favoured consistency of pain scores in both groups as all participants thought they were part of a study investigating the stability of measures over time. We found that pain intensity with hopping was greater in participants who received a traditional diagnostic explanation that included reference to tissue pathology. The point estimate of the effect size suggest that the content of diagnostic information has a moderate effect on pain intensity with loading. The upper end of the confidence interval includes values that might be regarded as representing a substantial between group difference, while the lower end of the confidence includes values that may be considered only a small effect.^25^ However, this interpretation is based on estimates of clinical significance for longitudinal clinical trials, where cost, time, effort, and risk are likely much greater than those associated with the intervention tested here.

The pre-intervention scores were near identical between groups and the post-intervention difference seems to be driven by both a worsening of scores in the tendon pathology group and a lessening of pain intensity in the functional explanation group. This finding is in accordance with Bayesian models of pain which contend that pain emerges as an interaction between sensory information, prior information held by the individual, and contextual cues.^1^^,^^6^^,^^27^ We explicitly asked about pain expectancy and saw little difference in this estimate between groups, potentially pointing to the importance of held modulatory information that exists outside of consciousness.^1^^,^^6^^,^^27^

Changes in stretch shortening cycle function followed a similar pattern, albeit with some uncertainty. Previous research on people with persistent AT suggests that decreased stiffness with loading is a consistent feature of the condition.^28^^,^^29^ While changes in tendon structure may contribute to this finding,^30^^,^^31^ the changes observed in the timescale of this study likely involve adjustments within the neuromotor system that drives the stretch shortening cycle.^32^^,^^33^ We found that those who received the traditional tendon pathology explanation had lower stiffness scores post-intervention, although the 95 % CI around this estimate crossed zero. The post-intervention difference reflects a decrease in stiffness in the tendon pathology group while there was a small increase in the functional explanation group. In line with previous suggestions^34^^,^^35^ these changes in stretch shortening cycle function might represent the behavioural correlate of decreased confidence in loading the tendon in those given the pathology orientated explanation. This finding also offers some tentative support for the idea that decreasing stiffness under stretch shortening load may be maladaptive, as this strategy was associated with higher pain intensity. While lower stiffness is a consistently observed feature of the condition, we know of no investigation that has tracked the trajectory to the low stiffness state. Indeed, from the current literature it is unclear if it is a risk factor for development of AT, a consequence of being in pain, both, or neither.^36^^,^^37^ These data suggest the possibility that a portion of the low stiffness state might develop post onset and reflect the participants understanding of the reason for their sensitivity state, however more data are required to assess this claim.

Our findings are consistent with previous research demonstrating a relationship between information about the state of the body and pain. Healthy pain-free individuals reported an increase in sensitivity to noxious stimulation when a body part was made to appear red and potentially ‘inflamed’.^15^ When healthy individuals embody an avatar of a virtual arm protected by wearing armour, they reported less pain with electrical stimulation than in conditions in which the embodied arm was not protected.^38^ Similar findings are seen in studies employing the rubber hand illusion, with sensitivity increasing if the embodied rubber hand is made to appear injured.^14^^,^^39^ In clinical settings, people with low back pain given a specific anatomically based diagnosis for their problem had worse outcomes than those given a non-specific explanation.^18^ Holding a more patho-anatomical view of low back pain is also associated with higher levels of disability.^16^ Unhelpful information regarding the structural integrity of the spine may also be behind the finding of an association between poor outcome and the provision of spinal imaging in those with low back pain,^40^ an idea reinforced by the observation that providing people with imaging reports that reference spinal pathology lead to worse outcomes.^17^

Our findings give some assurance that these impacts on clinical status might relate directly to the diagnostic information about the cause of pain provided to the individual as they were immediate and not confounded by subsequent actions taken by the individual or treatment provided. While other studies employing a randomised design have provided some evidence that diagnostic labels used to describe musculoskeletal pain problems have an immediate effect on shaping the views people hold regarding the severity of hypothetical clinical scenarios^11^ and their management^41^^,^^42^ to the authors’ knowledge, this is the first dataset demonstrating a possible causal link between diagnostic information and increased pain in a clinical population.

These findings need to be considered in light of the study limitations. As the sample size calculation was based on pain intensity only, the uncertainty around the adequacy of power to detect changes in leg stiffness and Time-to-Ease means that findings for analyses of secondary outcomes should be interpreted as exploratory. The clinician providing the explanation was not masked and this means performance bias may contribute to some of the effect estimates presented here. We tried to minimise this by extensive piloting of the delivery of the diagnostic explanations as well as use of a script to standardise information delivery. Performance bias was also minimised by ensuring that participants were masked. As the study was run at a single centre specialising in the management of lower limb tendinopathies and the same clinician gave all diagnostic explanations, this might limit the generalisability of our findings. Further research with larger samples, in different settings and with a variety of clinicians is needed to understand the generalisability of these results, as would the application of a similar methodology to different pain conditions. Finally, the design we employed enabled us to provide a relatively unbiased estimate of the immediate effects of diagnostic content, however, the ongoing influences are, as yet, unknown.

## Conclusions

The content of diagnostic information about the cause of pain influenced pain intensity with loading in a clinical pain population. Pain intensity was greater in participants who received a traditional diagnostic explanation that included reference to tissue pathology compared to people who received a diagnostic explanation that did not discuss tissue pathology. The effect on motor function was uncertain and there appeared to be no effect on the time it took for pain to ease.

## Declaration of competing interest

The authors declare no competing interest.

## Appendix Supplementary materials

Image, application 1
