# Supplementary material for: The content of diagnostic information has an immediate effect on pain with loading in people with midportion Achilles tendinopathy: a randomized clinical experiment
Source: Braz J Phys Ther. 2025 Jul 12;29(5):101244. doi: 10.1016/j.bjpt.2025.101244 (PMC12280333; doi:10.1016/j.bjpt.2025.101244)

Experimental Condition Script

Your tendon connects your muscles and bones. When a muscle contracts, the tendon transmits force to produce joint movement. Tendons are really stiff, much stiffer than muscles and they can withstand large loads with minimal deformation. With their strength, they can effectively transmit muscle force to produce movement. Tendons are described as being mechanically ‘stiff’. Tendon stiffness is this property of the tendon which enables us to be really springy, efficient and explosive when we run, hop and jump. Think of a kangaroo; they are the best at this!

[picture of athlete jumping]

Tendons are constructed of a type of material known as collagen. Collagen is made of very strong strands of tissue arranged along the line of pull of the tendon. Collagen is incredibly strong, up to ten times stronger than steel; as such, tendons are incredibly strong and the more collagen the tendon has the stronger it is. Interestingly, by targeted training, the body can increase the production of collagen and produce a thicker, stronger tendon. So just like muscles can get bigger and stronger with exercise, so can tendons. We can then get even more efficient in transmitting the force of our muscles to our bones so we can do amazing things like this!

[pictures of athlete jumping and picture of an athlete hopping]

In relation to your problem, I have had a good look at you and have a really good understanding of what is going on. It is very clear that the problem that you have is what is termed Achilles tendinopathy. Whilst the tendon is what is causing your pain, the good news is that there is no major structural damage to the tendon and the tendon ultimately remains strong.

The key to management of Achilles tendinopathy is addressing the factors that caused the problem in the first place. Interestingly, our understanding of the main factors driving tendinopathy have changed a lot in recent years. We used to think the problem was due simply to inflammation within the tendon, so lots of our treatments were directed there. However, we know that this is not the case; in fact, the evidence shows that there is not much wrong structurally with the tendon at all, which is great news for you.

What actually seems to be the main issue is a problem with the way the muscle that attaches to

the tendon is working. Basically, lack of muscle strength, ‘overloads’ the tendon which makes it sensitive.

For Achilles tendinopathy this is mainly the calf muscle but can also be due to issues in other muscles of the leg as they all work together to produce efficient and springy movement. The idea now is very clearly that the tendon is the victim of a problem elsewhere, in this case the calf and other leg muscles. The tendon is not the main culprit. We need to get the muscles of the lower leg working better so that load is distributed and shared more appropriately through the whole lower leg.

The tendon is in really good shape –we just need to get the muscles in good shape too.

[picture of athlete performing calf raises]

Control Condition Script

Your tendon connects your muscles and bones. When a muscle contracts, the tendon transmits force to produce joint movement. Tendons are really stiff, much stiffer than muscles and they can withstand large loads with minimal deformation. With their strength, they can effectively transmit muscle force to produce movement. Tendons are described as being mechanically ‘stiff’. Tendon stiffness is this property of the tendon which enables us to be really springy, efficient and explosive when we run, hop and jump.

[anatomical picture of Achilles tendon]

Tendons are constructed of a type of material known as collagen. Collagen is made of very strong strands of tissue arranged along the line of pull of the tendon. Collagen is incredibly strong, up to ten times stronger than steel; as such, tendons are incredibly strong and the more collagen the tendon has the stronger it is. Interestingly, by targeted training, the body can increase the production of collagen and produce a thicker, stronger tendon. So just like muscles can get bigger and stronger with exercise, so can tendons. We can then get even more efficient in transmitting the force of our muscles to our bones.

[cross sectional drawing of tendon structure]

In relation to your problem, I have had a good look at you and have a really good understanding

of what is going on. It is very clear that the problem that you have is what is termed Achilles tendinopathy. This diagnosis is quite broad and basically means that due to overload, the tendon has become painful.

The key thing to management of the problem is understanding why this is the case. While we used to think the problem was due to inflammation in the tendon, we now know this to be not entirely true. Actually, it appears that the condition is much more of a degenerative process rather than an inflammatory process in the tendon. The model that most people use when talking about Achilles tendinopathy describes a continuum of degeneration within the tendon with three stages.

The first stage is known as the Reactive Phase, which is brought about by sudden unexpected overload to the tendons. When the tendon is overloaded in this manner the tendon becomes a little swollen, like you see in this diagram:

[ultrasound image comparing normal tendon to reactive tendon]

The tendon usually recovers back to normal if the tendon is rested for a short period, such as 3-5 days. If it is not, the tendon can move to the next phase on the continuum.

The next phase is known as tendon ‘Disrepair’. In this phase, we see some breakdown of the normal tendon structure and disorganisation in the way collagen is arranged in the tendon. Also, for reasons we don’t fully understand, the tendon attempts to heal itself but doesn’t do a very good job of it.

The problem with this phase is that the collagen disorganisation affects the strength and stiffness of the tendon. The tendon structure when you look at it under a microscope is now disrupted and disorganised.

[photo of histological slide of tendon]

[ultrasound image tendon disrepair]

The final phase in the continuum is that of degenerative tendinopathy, which represents a failure of the tendon to repair properly. In this phase cells begin to die and there is further breakdown of the tendon due to further collagen breakdown. This most likely represents some irreversible tendon changes.

[ultrasound image illustraing degenerative tendinopathy]

The damaged area of the tendon gets filled with ingrowing blood vessels and by-products of tendon breakdown but unfortunately very little collagen.

It is thought that the sensitive area of the tendon is these areas of degeneration, where there is lack of collagen and excessive blood vessel ingrowth

[image demonstrating vascular ingrowth]

Boxplots for actual pain intensity, lower limb stiffness and time to ease post intervention.

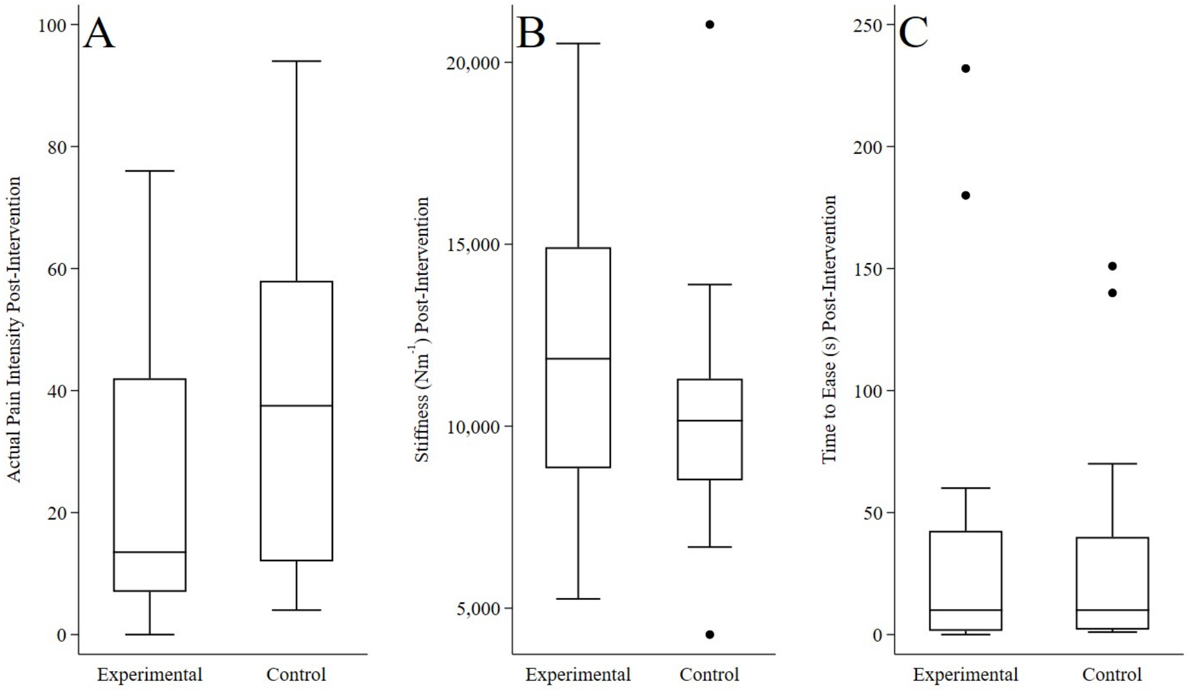

Supplement: Supplementary file 1 [file mmc1.pdf]
